# One-Step Synergistic SDS-H_2_O_2_ Process for High-Purity Chitin Extraction from Fly Larvae

**DOI:** 10.3390/polym17070994

**Published:** 2025-04-07

**Authors:** Yuhuan Qiu, Zhongtao Zhao, Feng Hu, Mengyi Liu, Xiaowen Shi

**Affiliations:** 1 School of Resource and Environmental Science, Wuhan University, Wuhan 430079, China; 2 Hubei Fisheries Science Institute, Wuhan 430077, China

**Keywords:** chitin, purification, sodium dodecyl sulfate, hydrogen peroxide solution

## Abstract

This study develops an efficient method for chitin extraction from fly larvae using a synergistic sodium dodecyl sulfate (SDS)-hydrogen peroxide (H_2_O_2_) system. Through a three-factor orthogonal experimental design, the optimal conditions were determined as 7% H_2_O_2_, 80 °C, and 15% SDS, achieving 97.93% deproteinization and 95.66% lipid removal efficiencies. Comparative analyses revealed that the SDS-H_2_O_2_ system outperformed traditional alkaline and deep eutectic solvent treatments in both purification performance and structural preservation. Fourier-transform infrared spectroscopy (FTIR) and X-ray diffraction (XRD) confirmed the high purity and crystallinity of the extracted chitin. Chitin prepared by this method demonstrated good Pb^2+^ adsorption (99.91%), highlighting its potential for targeted heavy metal remediation.

## 1. Introduction

Chitin, a naturally abundant amino-polysaccharide, has garnered considerable scientific interest owing to its extensive applications in biomedical and food industrial sectors [1,2,3]. Conventional chitin production mainly relies on crustacean exoskeletons obtained as seafood processing by-products [4,5,6,7]. Nevertheless, this approach faces critical limitations including seasonal supply instability and complicated extraction processes involving demineralization and deproteinization, which collectively contribute to elevated production costs and environmental burdens. Recent studies have increasingly focused on insect-derived chitin extraction, with particular attention to dipteran larvae due to their notable advantages in sustainable generation. Previous studies have shown that insects typically contain 3–10% chitin, 28–74% protein, 14–57% lipids, and 2–10% ash, making them a high-quality source of chitin extraction [8,9,10]. Furthermore, chitin derived from insect sources demonstrates better antimicrobial activity and antioxidant properties relative to crustacean-derived chitin [11,12,13,14].

Chemical and biological methods are commonly used for extracting insect chitin. Chemical methods generally involve four steps, namely, demineralization, deproteinization, degreasing, and decolorization. In the demineralization process, acidic solutions such as HCl and HAc are generally employed. For deproteinization, alkaline solutions like NaOH and KOH are typically utilized. Degreasing generally relies on organic solvents, while decolorization relies on oxidizing agents [12,15,16,17]. Chemical methods demonstrate effective purification efficacy, achieving over 70% elimination of proteins and lipids [18,19]. However, these approaches can damage the structure of chitin and cause serious environmental pollution. Recent studies propose deep eutectic solvents (DESs) as eco-friendly alternatives due to their low toxicity, biodegradability, and high stability. Huet et al. achieved chitin isolation from silkworm and black soldier fly larvae using choline chloride and lactic acid (1:2 molar ratio) at 110 °C, reducing protein content from 45.8% to 34.1% and 60.3% to 52.2%, respectively [20]. Nevertheless, DES methods face limitations including incomplete impurity removal, solvent recovery challenges, and formulation optimization requirements [21]. Biological methods typically use enzymes or microbial fermentation. Silva Lucas et al. achieved 85% protein removal from mealworm by using *Bacillus licheniformis* lytic enzymes [22]. However, biological methods require longer reaction times and it is difficult to produce high-purity chitin.

Briefly, current chitin extraction methods from insects either use reagents with environmental pollution and biotoxicity, or demonstrate constrained impurity removal efficiencies. Moreover, most of these methods have separate steps, leaving room for improvement in procedural simplicity. Sodium dodecyl sulfate (SDS), an anionic surfactant, serves as a common protein denaturant while also functioning as a detergent with lipid removal capability. Hydrogen peroxide (H_2_O_2_), a widely used decolorizing agent, has been shown to be able to enhance the removal of residual protein in chitosan by SDS through increasing material porosity [23]. On these foundations, SDS and H_2_O_2_ together were chosen for the treatment of fly larvae with high protein and fat content. This study introduced a single-step extraction of chitin from fly larvae through the synergistic combination of SDS and H_2_O_2_, achieving simultaneous deproteinization, degreasing, and decolorization. H_2_O_2_ oxidized the pigments by generating hydroxyl radicals, which also broke the glycosidic bond of chitin and enlarged the pore size. In addition, the gas release due to the decomposition of H_2_O_2_ made the outer fat layer fluffier, facilitating the penetration of SDS into the interior structure of the larvae. SDS disrupted the spatial structure of protein through hydrophobic interaction, interfered with its hydrogen bonds with chitin, and combined with protein to form soluble complexes through electrostatic interactions. Meanwhile, SDS binds to water through its hydrophilic head and to lipids through its hydrophobic long chain, forming emulsions. Proteins and lipids were eventually removed by filtration. In the operating process, raw fly larvae with protein content over 67% and lipid content over 25% were treated with a mixture of SDS-H_2_O_2_ under stirring and heating. After this one-pot treatment, the residue was washed and dried to obtain high-purity chitin. A three-factor orthogonal experimental design was employed to quantify the effects of SDS concentration (9–15%), H_2_O_2_ concentration (3–7%), and processing temperature (60–80 °C) on purification efficiency. We found that under the conditions of 15% sodium dodecyl sulfate, 7% hydrogen peroxide, and 80 °C, the purity of chitin exceeded 90%, the protein content was reduced to 1.39% and that of lipids to 1.10%. We believe that this study has great potential for the large-scale extraction of chitin from fly larvae.

## 2. Materials and Methods

### 2.1. Materials

Fly larvae (*Musca domestica* L.) were supplied by Xijiruo Biochemical Co., Shanghai, China. Sodium dodecyl sulfate, hydrogen peroxide solution, sodium hydroxide, hydrochloric acid, sodium carbonate, copper sulfate pentahydrate, lead nitrate, cadmium sulfate 8/3 hydrate, zinc sulfate heptahydrate, petroleum ether, and malonate were purchased from Sinopharm Chemical Reagent Co., Shanghai, China. Folin phenol was purchased from Yuanye Biotechnology, Shanghai, China. Choline chloride was purchased from Macklin Biochemical Co., Shanghai, China. Potassium sodium tartrate and commercial-grade chitin were purchased from Aladdin Biochemical Technology Co., Shanghai, China. All reagents are of analytical grade without further purification.

### 2.2. Preparation of Chitin

**Figure 1:**
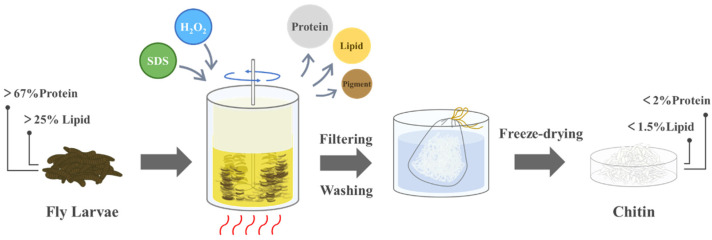
Extraction process of chitin from fly larvae through SDS-H_2_O_2_ treatment.

The preparation process is shown in Figure 1. In each group, 20.00 g of fly larvae was treated with 250 mL SDS-H_2_O_2_ mixed water solution under constant-temperature water bath agitation for 6 h. The resulting product was filtered through a mesh bag, thoroughly washed with distilled water, and freeze-dried for 12 h. The specific experimental parameters are presented in Section 2.3 Orthogonal experimental design. The yield was calculated as follows:

Yield (%) = (M_c_/5.00) × 100% (1)

In the formula, 5.00 is the converted dry weight (g) of 20 g fly larvae raw material, and M_c_ is the mass of chitin produced.

### 2.3. Orthogonal Experimental Design

The experimental parameters were determined based on a three-factor, two-level L8 orthogonal array design, with the specific factor levels established during preliminary experiments, as detailed in Table 1.

Following the orthogonal experimental design, eight experiments were implemented, with the detailed operational parameters presented in Table 2.

### 2.4. Control Experimental Design

The control experimental procedures comprised four distinct treatments. For the SDS treatment (designated as Sample S15), 20.00 g of fly larvae was mixed with 250 mL of 15 wt% SDS solution and incubated at 80 °C for 6 h. In the hydrogen peroxide treatment (Sample H7), an equivalent mass of fly larvae (20.00 g) was treated with 250 mL of 7 wt% H_2_O_2_ solution under identical thermal conditions (80 °C for 6 h). For the alkali treatment (Sample Alk), 20.00 g of fly larvae was combined with 50 mL of 1 mol/L NaOH and heated in a 95 °C water bath for 6 h [12]. The choline chloride–malonic acid treatment (Sample CCMA) involved drying and pulverizing fly larvae to obtain a powdered form. Subsequently, 5.00 g of the dried powder was homogenized with 35.00 g of a choline chloride–malonic acid deep eutectic solvent (1:2 molar ratio), followed by continuous stirring in a 50 °C water bath for 6 h [24].

### 2.5. Protein Content Measurement

Protein content was determined via Lowry’s method [25]. A chromogenic reaction of the standard substance was firstly performed to determine its spectrum in the wavelength range of 450–780 nm. The maximum absorption peak at 750 nm was selected as the wavelength for absorbance measurement. Samples (0.20 g) were digested in 20 mL 5% NaOH at 95 °C for 2 h [26,27]. The pH-adjusted supernatant (10.00) was reacted with Reagent A (alkaline copper solution) for 10 min, followed by Reagent B (Folin phenol). Absorbance at 750 nm was measured after 30 min using a Shimadzu UV-1780. The standard curve was plotted through bovine serum albumin (BSA) solution to calibrate the protein content in chitin. The protein content and deproteinization efficiency were calculated as follows:

Protein Content (wt%) = [(P_s_ × V_s_)/m_s_] × 100% (2)

In the formula, P_s_ represents the measured protein content of the supernatant (mg/mL), V_s_ represents the final volume of the supernatant after heating and pH-adjusting (mL), and m_s_ is the mass of the sample used for measurement (mg).

Deproteinization Efficiency (%) = [(P_l_ − P_c_)/P_c_] × 100% (3)

In the formula, P_l_ represents the protein content of the raw larvae (%), and P_c_ represents the protein content of the chitin sample (%).

### 2.6. Lipid Content Measurement

Lipid content was measured by Soxhlet extraction [8,10]. Pre-weighed dried samples were wrapped in filter paper and placed in the extraction chamber. Petroleum ether solvent circulated through the system at ≥10 cycles/hour for 6 h (8 h for high-lipid samples). The extracted lipids were collected in flasks, dried at 105 °C, and weighed until constant mass was obtained. The lipid content and degreasing efficiency were calculated as follows:

Lipid content (wt%) = [(W_fl_ − W_f_)/M_ds_] × 100% (4)

In the formula, M_ds_ is the mass of the dried sample used for defatting (g), W_f_ is the initial weight of the flask (g), and W_fl_ is the weight of the flask mass containing extracted lipids (g).

Degreasing Efficiency (%) = [(L_l_ − L_c_)/L_c_] × 100% (5)

In the formula, L_l_ represents the lipid content of the raw larvae (%), and L_c_ represents the lipid content of the chitin sample (%).

### 2.7. Ash and Moisture Measurement

Moisture content was determined using the drying method. Samples were oven-dried at 105 °C for 24 h following initial mass measurement. Moisture content was calculated as:

Moisture content (wt%) = [(M_bd_ − M_ad_)/M_bd_] × 100% (6)

In the formula, M_bd_ represents the sample mass before drying (g), and M_ad_ represents the sample mass after drying (g).

Ash content was determined using the combustion method. Dried samples were combusted in pre-weighed crucibles at 700 °C for 6 h [28,29] using a tube furnace. Ash content was determined by:

Ash content (wt%) = [(M_s_ − W_cs_ + W_c_)/M_s_] × 100% (7)

In the formula, M_ds_ represents the mass of the sample (g), W_c_ represents the weight of the empty crucible (g), and W_cs_ represents the total weight of the crucible and sample after burning (g).

### 2.8. Purity Measurement

The chitin content (wt%) of the raw larvae (dried) was calculated by:

Chitin content (wt%) = 100% − P_l_ − L_l_ − A_l_ (8)

In the formula, P_l_ is the protein content of the raw larvae (wt%), L_l_ is the lipid content of the raw larvae (wt%), and A_l_ is the ash content of the raw larvae (wt%).

For the samples prepared in orthogonal experiments and control, the purity of chitin was calculated as:

Purity (%) = [C_cl_ × M_l_/M_ts_] × 100% (9)

In the formula, C_cl_ is the chitin content of the raw larvae (%), M_l_ is the mass of the raw larvae for chitin extraction (g), and M_ts_ is the total mass of obtained chitin after treatment (g).

### 2.9. Characterizations

Chemical structures were determined by Fourier-transform infrared (FTIR) analysis. The samples were mixed with potassium bromide at a ratio of 1:100 and ground up, then pressed into tablets for determination. The infrared spectrum was recorded within the wavelength range of 4000 to 400 cm^−1^ using an FTIR spectrometer (NICOLET 5700 FTIR Spectrometer, Thermo Fisher Scientific, Waltham, MA, USA). Crystallographic structures were determined by X-ray diffraction (XRD) using MiniFlex 600 (RIKEN Electric Co., Tokyo, Japan) within the 2θ range of 6° to 60° with a scanning rate of 5°/min. The crystallization index (CrI) was calculated as follows:

Crystallization index (%) = [(I_100_ − I_am_)/I_100_] × 100% (10)

where I_100_ is the maximum intensity at 19° and I_am_ is the intensity of amorphous diffraction at 13°.

Thermogravimetry analyses (TGAs) were performed by STA7300 (Hitachi Instruments Co., Chiyoda, Japan) at a temperature ramping of 10 °C/min from 30 °C to 600 °C under a nitrogen atmosphere. Morphologic structures were observed using a tungsten filament scanning electron microscope (Tescan VEGA Compact, TESCAN ORSAY HOLDING, Brno, Czech Republic). The samples were gold-plated through a sputter coater (Quorum 150R Plus, Quorum Technologies, Inc., Lewes, UK) before observation.

### 2.10. Heavy Metal Adsorption Capacity

The heavy metal adsorption capacity was evaluated through a standardized experimental protocol. Stock solutions containing 200 mg/L [30] of Cu^2+^, Cd^2+^, Pb^2+^, and Zn^2+^ were prepared using CuSO_4_·5H_2_O, CdSO_4_·8/3H_2_O, Pb(NO_3_)_2_, and ZnSO_4_·7H_2_O, respectively. The prepared chitin samples were combined with the heavy metal solutions at a mass-to-volume ratio of 1:50 (m:v). The mixtures were agitated continuously at a temperature of 25 °C for 24 h, followed by filtration through 0.45 μm membrane filters. Residual metal ion concentrations in the filtrate were quantified using an inductively coupled plasma optical emission spectrometer (Agilent Technologies, Inc., Santa Clara, CA, USA). The monitored spectral lines of Cu, Cd, Pb, and Zn were 327.395 nm, 226.502 nm, 220.353 nm and 213.857 nm. Spectral backgrounds were corrected, and elemental characteristic lines were calibrated before the intensity of the elemental characteristic lines was measured.

The metal adsorption efficiency was calculated using the following formula:

η = [(C_0_ − C_e_)/C_0_] × 100% (11)

where η represents the adsorption efficiency (%), C_0_ denotes the initial metal ion concentration (mg/L), and C_e_ indicates the equilibrium concentration (mg/L) after treatment.

## 3. Results and Discussion

### 3.1. Orthogonal Experimental Results

The preparation conditions were explored by performing orthogonal experiments. The results of the orthogonal experiment are shown in Table 3. The numbers in the names of the experimental groups represent different SDS concentrations (9% and 15%), H_2_O_2_ concentrations (3% and 7%), and temperatures (70 °C and 80 °C).

The orthogonal experiment demonstrates that the SDS-H_2_O_2_ synergistic system significantly influences protein removal efficiency. Increasing H_2_O_2_ concentration from 3% to 7% enhanced deproteination rates from 61.73–88.16% to 91.85–97.93% (in the groups S9H3-70, S9H7-80, S15H3-70, and S15H7-80), confirming H_2_O_2_’s critical role in disrupting larval surface barriers and facilitating SDS-protein interactions. Elevated temperature (from 70 °C to 80 °C) further amplified this synergy. At 7% H_2_O_2_ concentration, the deproteination efficiency of the 80 °C treatment groups (S9H7-80: 97.38%; S15H7-80: 97.93%) was significantly higher than the 70 °C groups (S9H7-70: 95.00%; S15H7-70: 91.85%). This may be attributed to the thermally accelerated decomposition of H_2_O_2_, which amplifies its surface-disruptive effects, while elevated temperatures concurrently enhance SDS–protein binding interactions [23].

In general, the yield decreased and the purity increased as more impurities were removed. When the yield was less than 6.2%, the purity rose to over 80%. Notably, increasing SDS concentration from 9% to 15% showed limited improvement in deproteination efficiency. This suggests that the 9% SDS concentration achieves a good protein removal effect, and further increasing the SDS concentration will gradually reduce the benefits.

The range analysis (Tables S1 and S2) identified H_2_O_2_ concentration as the dominant factor (R = 25.19 for protein content and R = 50.64 for chitin purity), followed by temperature (R = 21.63 for protein content and R = 44.96 for chitin purity), with SDS concentration showing negligible effects (R = 1.87 for protein content and R = 0.85 for chitin purity). Optimal performance occurred at 7% H_2_O_2_, 80 °C, and 15% SDS (S15H7-80 group), yielding peak deproteinization (97.93%) and purity (91.15%).

### 3.2. Purification Performance

**Figure 2:**
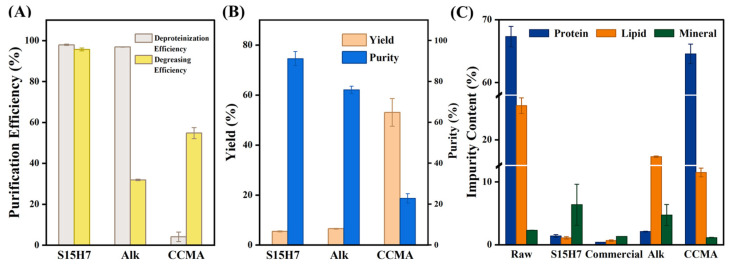
(**A**) Purification efficiency of chitin samples. (**B**) Yield and purity of chitin samples. (**C**) Component analysis of chitin samples.

The purification efficiencies of the tested samples are summarized in Figure 2. As shown in Figure 2A, the SDS-H_2_O_2_ optimized sample (S15H7) demonstrated superior integrated purification performance, achieving 97.93% deproteinization and 95.66% degreasing efficiency. These results significantly outperformed alkaline-treated (Alk) and deep eutectic solvent-treated (CCMA) chitin samples, particularly in lipid removal (only 31.90% for Alk). The S15H7 sample also performed better than previous reports on insect-based chitin purification (Figure 2B). For instance, Hahn et al. [31] reported 85% chitin purity in black soldier fly through acid demineralization, alkaline deproteinization, and decolorization treatments, while Huet et al. [20] reported chitin purity values of 60.2% and 42.8% for *Bombyx eri* and *Hermetia illucens* larvae with deep eutectic solvents. The variation in yield was negatively related to purity among S15H7, Alk, and CCMA. The chitin content of raw larvae was determined to be 4.96%, according to which the chitin yields of S15H7 (5.45%) and Alk (6.54%) were close to the theoretical content of pure chitin contained. This confirmed the removal of impurities. In contrast, CCMA showed a 53.07% yield, indicating a high content of impurities.

As shown in Figure 2C, the raw material exhibited high initial protein (67.32%) and lipid (25.42%) contents. Treatment with the SDS-H_2_O_2_ system significantly reduced these values to 1.39% protein and 1.10% lipid, outperforming Alk samples (2.09% protein and 17.31% lipid). In contrast, the deep eutectic solvent-treated group (CCMA) showed minimal deproteinization efficacy, retaining 64.57% protein content. Notably, the lipid content of S15H7 sample was close to the commercial chitin sample (0.65%), and this superior lipid removal can be attributed to the action of SDS. As an amphiphilic surfactant, SDS selectively binds to lipids through hydrophobic interactions while its hydrophilic groups facilitate solubilization in aqueous media [32]. With regard to the ash content, both S15H7 and Alk samples exhibited increased mineral residue compared to raw materials, which was an increase in the relative content attributed to the removal of proteins and lipids. In comparison, CCMA showed reduced ash content. This divergence stems from malonic acid, which is a well-established demineralization agent.

**Figure 3:**
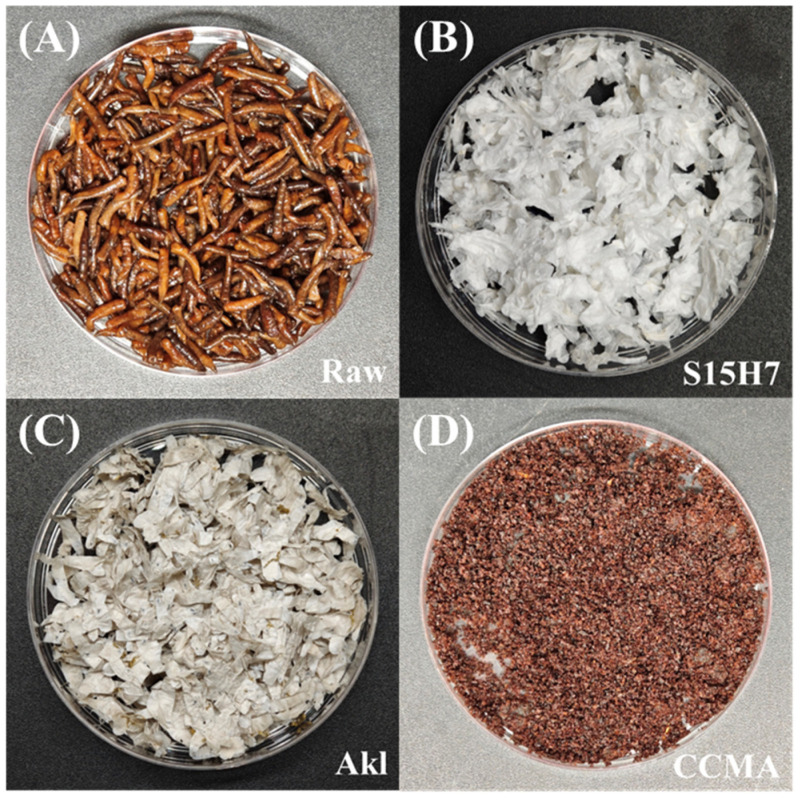
The visual characteristics of the (**A**) raw material, (**B**) S15H7, (**C**) Akl, and (**D**) CCMA.

Figure 3 displays the visual characteristics of the raw material (Figure 3A), S15H7 (Figure 3B), Alk (Figure 3C), and CCMA (Figure 3D) samples. The raw material and CCMA exhibited dark brown coloration, while Alk appeared off-white with a yellowish tint. In contrast, S15H7 was pure white. These color variations might correlate with the contents of viscera, lipid, and pigments, as the color became lighter when the impurities decreased. S15H7 had the best decolorization effect not only because it had the fewest impurities, but also due to the decomposition and removal of melanin in the epidermis and carotenoids in lipids by the oxidation of H_2_O_2_ [33,34].

**Figure 4:**
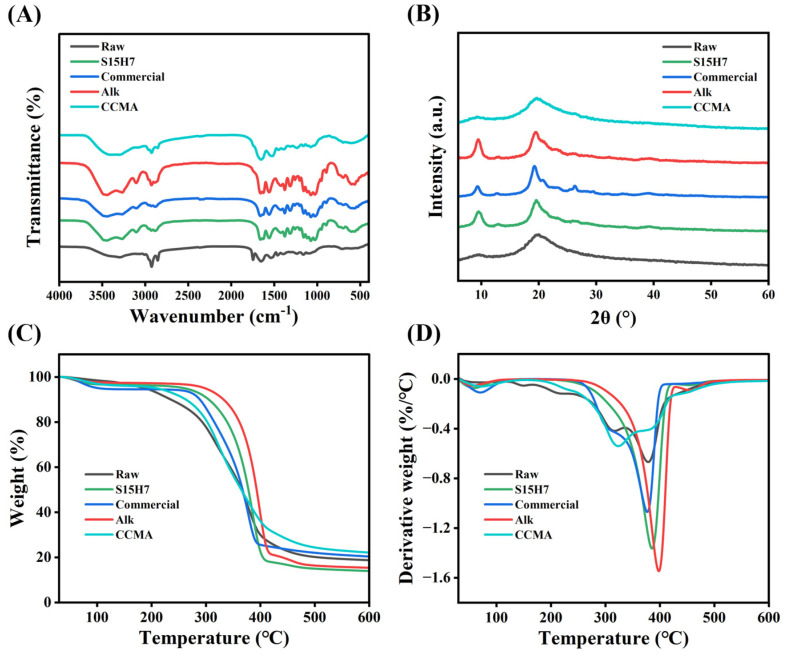
(**A**) FTIR spectra, (**B**) XRD curves, (**C**) TGA curves, and (**D**) DTG curves of Raw, S15H7, Commercial, Alk, and CCMA.

The FTIR spectra and XRD diffractograms of five samples (Raw, S15H7, Commercial chitin, Alk, and CCMA) are presented in Figure 4. In the FTIR analysis (Figure 4A), the Raw and CCMA samples exhibited numerous extraneous peaks across the 1500–3500 cm^−1^ range, indicative of residual proteins and lipids. In contrast, S15H7 and Commercial chitin displayed nearly identical characteristic chitin bands, namely, 3263 cm^−1^ (N-H stretching), 1654 cm^−1^ (amide I), 1558 cm^−1^ (amide II), and 1070 cm^−1^ (C-O-C bridge, C-O stretching, and C-C stretching), confirming successful purification [35,36,37,38]. The wide peaks around 3450 cm^−1^ corresponded to O-H stretching, which was from the OH group on chitin and possibly the adsorbed water [39] due to the hygroscopicity of chitin. Notably, Alk as well as Raw exhibited significantly intensified absorption peaks at 2929 cm^−1^ compared to S15H7 and Commercial chitin. This characteristic peak corresponded to CH_3_ symmetric stretching and CH_2_ asymmetric stretching vibrations, attributable to residual lipid in the sample. In addition, Raw exhibited a significant peak at 1747 cm^−1^, corresponding to C=O that belongs to esters [40], while S15H7 and Commercial exhibited smooth lines, demonstrating the effective degreasing.

The XRD spectra (Figure 4B) revealed distinct crystallinity variations. The Raw and CCMA samples showed broad, low-intensity peaks at 2θ = 9.4° and 19.3°, characteristic of amorphous biopolymer mixtures. In contrast, S15H7 and Alk displayed sharp crystalline reflections at 2θ = 9.3° and 19.1°, aligning with the characteristic of α-chitin [41,42]. The crystallization indices (CrI) of S15H7, Alk, and Commercial were 76.30%, 76.69%, and 87.08%.

The composition and thermal stability were further analyzed through TGA (Figure 4C,D). The initial mass loss stage (30–170 °C) in all curves corresponded to moisture evaporation [43], while the subsequent mass loss stage (170–600 °C) was attributed to the decomposition of proteins, lipids, and chitin. Notably, Raw and CCMA exhibited earlier decomposition onsets and more pronounced mass loss within the 170–250 °C range, corresponding to protein and lipid degradation. The primary mass loss regions for Commercial, S15H7, and Alk samples spanned 240–405 °C, 212–420 °C, and 216–428 °C while Raw and CCMA showed extended decomposition processes persisting until about 510 °C (Figure 4D), resulting from the continuous breakdown of residual proteins and lipids [44]. These results confirmed the effective removal of proteins and lipids in S15H7 and Alk. The total mass losses of Commercial, S15H7, and Alk were 79.62%, 84.61%, and 86.04%. The maximum decomposition rate temperatures (DTG_max_) were compared as follows: Commercial (377 °C) < S15H7 (385 °C) < Alk (398 °C). In brief, chitin derived from house fly larvae exhibited comparable thermal stability to commercial chitin.

### 3.3. Purification Mechanism

**Figure 5:**
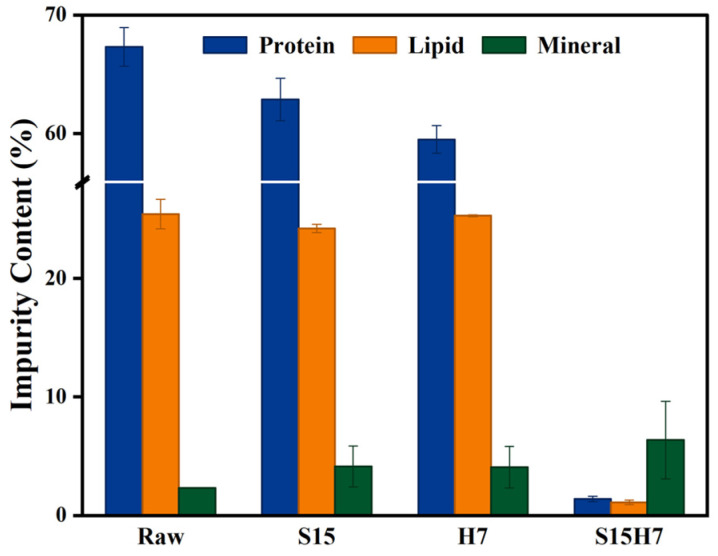
The impurity content of each experimental group.

In order to investigate the mechanism of SDS-H_2_O_2_ deproteinization, experimental groups were prepared using only 15% SDS (S15) and only 7% H_2_O_2_ (H7), with other reaction conditions consistent with 15% SDS, 7% H_2_O_2_, and 80 °C (S15H7). The impurity content of each experimental group is shown in Figure 5.

As shown in Figure 5, the SDS-H_2_O_2_ synergistic system (S15H7) achieved superior impurity removal, compared to individual SDS or H_2_O_2_ treatments. As for S15H7, the protein content was reduced from Raw’s 67.32% to 1.39%, and the fat content was reduced from 25.42% to 1.10%, while there remained 62.86% protein residue and 24.22% lipid residue in S15, and 59.50% protein residue and 25.29% lipid residue in H7. These results demonstrated that the enhanced deproteinization efficacy of S15H7 originates from the synergistic interaction between SDS and H_2_O_2_. SDS, as an anionic surfactant, solubilizes proteins through hydrophobic interactions and emulsifies lipids through micelle formation, while H_2_O_2_ is an oxidant that disrupts the surface structure, increasing porosity and enhancing SDS penetration. This result is consistent with findings by Zhao et al. [23], who reported that H_2_O_2_ enhances protein removal in chitosan by generating microporous structures.

**Figure 6:**
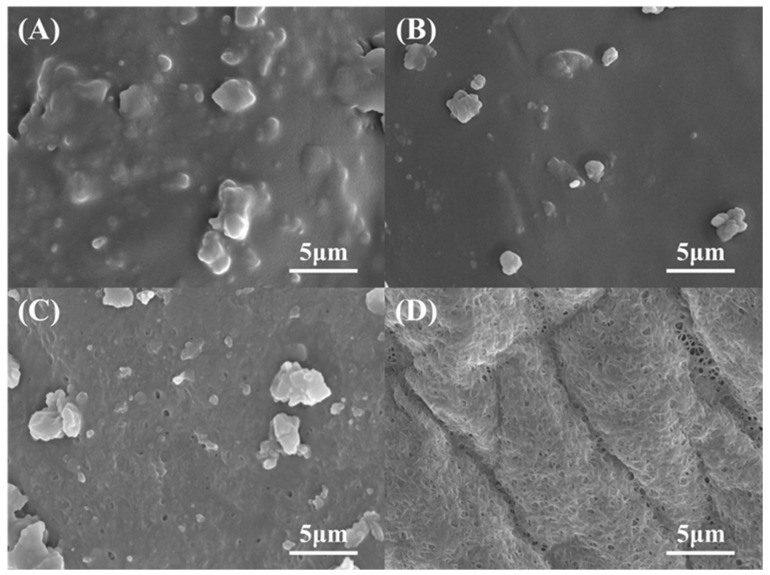
SEM images of (**A**) raw sample, (**B**) S15, (**C**) H7, and (**D**) S15H7.

This synergistic effect was further confirmed by SEM observation. The raw samples (Figure 6A) displayed rough surfaces with protrusions, corresponding to protein–lipid complexes. SDS-only treatment (B) partially smoothed the surface by solubilizing proteins and lipids on the surface, while H_2_O_2_-alone treatment (C) produced minor pores. Remarkably, the SDS-H_2_O_2_ synergy (D) generated a densely porous architecture devoid of protrusions, indicating the removal of impurities.

Based on these observations, the synergistic mechanism of the SDS-H_2_O_2_ system can be elucidated as follows. H_2_O_2_ first creates structural pores through oxidation and then SDS penetrates these pores to solubilize and remove proteins and lipids. This two-stage process explains the synergistic effect of SDS and hydrogen peroxide, which has higher performance than individual treatments.

### 3.4. Heavy Metal Adsorption Performance

Chitin is recognized as a competent adsorbent for toxic metals due to its pore structure and groups (-NH_2_, -OH). Although its modifiability is comparatively lower than that of its derivative chitosan, chitin is more economical [45]. The heavy metal adsorption capacities of S15H7-80, Alk, and Commercial samples were comparatively evaluated (Table 4).

The Alk-treated sample demonstrated superior adsorption efficiency for Cd^2+^ (76.11%), Cu^2+^ (94.00%), and Zn^2+^ (72.83%), outperforming both S15H7-80 and Commercial samples. This enhanced performance may be due to the fact that the alkaline treatment leads to the partial deacetylation of chitin. Notably, S15H7-80 achieved exceptional Pb^2+^ adsorption (99.91%). Forutan et al. documented a maximum Pb^2+^ adsorption of 7.003 mg/g using pink shrimp derived chitin, lower than the 9.991 mg/g achieved by S15H7-80 in this study [46]. These results suggest its potential application in lead-specific remediation. The order of adsorption capacity of S15H7-80 for the four ions was Pb^2+^ > Cu^2+^ > Cd^2+^ > Zn^2+^, consistent with the studies of Rhazi et al. [47] and Wang et al. [30]. The high adsorption capacity for Pb^2+^ was caused by the strong chelation between Pb^2+^ and amino (-NH_2_) and hydroxyl (-OH) groups on chitin.

## 4. Conclusions

This study established an efficient method for chitin extraction from fly larvae using a synergistic SDS-H_2_O_2_ system. Orthogonal experimental results demonstrated that H_2_O_2_ concentration and temperature were the dominant factors influencing purification efficiency, with optimal conditions identified as 7% H_2_O_2_, 80 °C, and 15% SDS. Under these parameters, the system achieved 97.93% deproteinization and 95.66% lipid removal with 5.45% yield and 91.15% purity, significantly outperforming traditional alkaline and deep eutectic solvent treatments. Structural characterization via FTIR and XRD confirmed the high purity and crystallinity of the extracted chitin.

The synergistic mechanism was elucidated through comparative experiments and SEM analysis. The H_2_O_2_ disrupted surface barriers to enhance SDS penetration, while SDS effectively solubilized proteins and emulsified lipids. Chitin prepared by this method demonstrated good Pb^2+^ adsorption (99.91%), highlighting its potential for targeted heavy metal remediation.

This single-step strategy eliminates the need for harsh alkaline conditions, reduces energy consumption, and minimizes environmental impact. The findings provide a scalable, sustainable alternative to conventional crustacean-based chitin production. Future research should focus on process optimization for industrial-scale applications.

## Figures and Tables

**Table 1:** Orthogonal experimental design.

Parameter	Level	
	1	2
SDS (wt%)	9	15
H_2_O_2_ (wt%)	3	7
Temperature (℃)	70	80

**Table 2:** Specific parameters of 8 experimental groups.

Experimental Group	SDS (wt%)	H_2_O_2_ (wt%)	Temperature (°C)
S9H3-70	9	3	70
S9H3-80	9	3	80
S9H7-70	9	7	70
S9H7-80	9	7	80
S15H3-70	15	3	70
S15H3-80	15	3	80
S15H7-70	15	7	70
S15H7-80	15	7	80

**Table 3:** Orthogonal experimental results.

Experimental Group	Yield (%)	Protein (%)	Deproteinization Efficiency (%)	Purity of Chitin (%)
S9H3-70	12.47 ± 0.42	25.76 ± 2.59	61.73 ± 3.85	39.82 ± 1.32
S9H7-70	5.84 ± 0.18	3.36 ± 1.11	95.00 ± 1.64	84.99 ± 2.69
S15H3-70	11.07 ± 1.01	24.2 ± 1.88	64.05 ± 2.8	45.08 ± 4.35
S15H7-70	6.16 ± 0.12	5.49 ± 1.32	91.85 ± 1.96	80.54 ± 1.65
S9H3-80	6.05 ± 0.19	4.44 ± 1.07	93.41 ± 1.59	82.08 ± 2.58
S9H7-80	5.55 ± 0.10	1.76 ± 0.52	97.38 ± 0.77	89.34 ± 1.66
S15H3-80	6.38 ± 0.14	7.97 ± 0.73	88.16 ± 1.09	77.77 ± 1.67
S15H7-80	5.45 ± 0.21	1.39 ± 0.22	97.93 ± 0.33	91.15 ± 3.48

**Table 4:** Heavy metal adsorption rates (%).

Metal Ion	Adsorption Rate (%)		
	S15H7-80	Alk	Commercial
Cd^2+^	35.05 ± 2.89	76.11 ± 1.28	43.03 ± 8.64
Cu^2+^	46.02 ± 0.63	94.00 ± 0.07	81.15 ± 0.08
Pb^2+^	99.91 ± 0.04	99.63 ± 0.10	96.04 ± 0.35
Zn^2+^	52.47 ± 4.40	72.83 ± 1.15	34.64 ± 5.78

## Data Availability

The original contributions presented in the study are included in the article/Supplementary Material, further inquiries can be directed to the corresponding author.

## Supplementary Materials

The following supporting information can be downloaded at: https://www.mdpi.com/article/10.3390/polym17070994/s1, Table S1: Range analysis for protein content; Table S2: Range analysis for purity of chitin.
