# Supplementary material for: One-Step Synergistic SDS-H2O2 Process for High-Purity Chitin Extraction from Fly Larvae
Source: Polymers (Basel). 2025 Apr 7;17(7):994. doi: 10.3390/polym17070994 (PMC11991004; doi:10.3390/polym17070994)
Supplement: Supplementary file 1 [file polymers-17-00994-s001.zip › polymers-3563795-SI.pdf]

Supporting Information

One-Step Synergistic SDS-H_2O_2 Process for High-Purity Chitin Extraction from Fly Larvae

Yuhuan Qiu [1,†], Zhongtao Zhao [2,†], Feng Hu [1], Mengyi Liu [1] and Xiaowen Shi [1,*]

[1] School of Resource and Environmental Science, Wuhan University, Wuhan 430079, China
[2] Hubei Fisheries Science Institute, Wuhan 430077, China
* Correspondence: shixw@whu.edu.cn
† These authors contributed equally to this work.

Table S1 Range analysis for protein content

Experimental Group	Preparation Condition						Protein Content(%)
	**SDS**	**H_2O_2**	**SDS-H_2O_2**	**T**	**SDS-T**	**H_2O_2-T**	
S9H3-70	1	1	1	1	1	1	25.76±2.59
S9H3-80	1	1	1	2	2	2	4.44±1.07
S9H7-70	1	2	2	1	1	2	3.36±1.11
S9H7-80	1	2	2	2	2	1	1.76±0.52
S15H3-70	2	1	2	1	2	2	24.20±1.88
S15H3-80	2	1	2	2	1	1	7.97±0.73
S15H7-70	2	2	1	1	2	1	5.49±1.32
S15H7-80	2	2	1	2	1	2	1.39±0.22
K1	35.32	62.37	37.08	58.81	38.48	40.98	
K2	39.05	12.00	37.29	15.56	35.89	33.39	
k1	17.66	31.19	18.54	29.41	19.24	20.49	
k2	19.53	6.00	18.65	7.78	17.95	16.70	
**R**	1.87	25.19	0.11	21.63	1.30	3.80	

Table S2 Range analysis for purity of chitin

Experimental Group	Preparation Condition						Purity Of Chitin(%)
	**SDS**	**H_2O_2**	**SDS-H_2O_2**	**T**	**SDS-T**	**H_2O_2-T**	
S9H3-70	1	1	1	1	1	1	39.82±1.32
S9H3-80	1	1	1	2	2	2	82.08±2.58
S9H7-70	1	2	2	1	1	2	84.99±2.69
S9H7-80	1	2	2	2	2	1	89.34±1.66
S15H3-70	2	1	2	1	2	2	45.08±4.35
S15H3-80	2	1	2	2	1	1	77.77±1.67
S15H7-70	2	2	1	1	2	1	80.54±1.65
S15H7-80	2	2	1	2	1	2	91.15±3.48
K1	296.23	244.75	293.59	250.43	293.73	287.47	
K2	294.54	346.02	297.18	340.34	297.04	303.3	
k1	148.12	122.38	146.80	125.22	146.87	143.74	
k2	147.27	173.01	148.59	170.17	148.52	151.65	
**R**	0.845	50.64	1.795	44.96	1.66	7.92	
